# Dental Students’ Temporomandibular Disorder‐Related Education, Knowledge, Attitudes, and Behavior, How Do Personal Experiences Matter?

**DOI:** 10.1002/jdd.70008

**Published:** 2025-08-21

**Authors:** Monica Fan, Elizabeth R. Hatfield, Shruti K. Sivakumar, Marita R. Inglehart

**Affiliations:** ^1^ University of Michigan School of Dentistry Ann Arbor Michigan USA; ^2^ Department of Oral and Maxillofacial Surgery University of Michigan School of Dentistry Ann Arbor Michigan USA; ^3^ Department of Periodontics and Oral Medicine Department of Psychology School of Dentistry College of Literature University of Michigan Ann Arbor Michigan USA

**Keywords:** dental education, dental schools, dental students, dentists, temporomandibular joint disorders, temporomandibular joint, temporomandibular disorders

## Abstract

**Objectives:**

Approximately, 5%–12% of adults in the United States are estimated to have temporomandibular disorders (TMD), making it crucial that dentists are optimally trained to diagnose and treat TMD. The objectives were to assess dental students’ TMD‐related education, knowledge, attitudes, and behavior and explore if personal TMD‐related experiences were associated with constructs of interest.

**Methods:**

A total of 469 dental students participated in this survey‐based cross‐sectional study by reporting personal TMD experiences, TMD‐related knowledge, attitudes, and professional behaviors. Factor analyses were used to create indices for constructs of interest. Hypotheses about the relationships between these constructs were tested in correlational analysis.

**Results:**

Approximately, 29.5% were satisfied with their TMD‐related dental school education, 49.9% felt well‐prepared to diagnose TMD in classroom, and 35.6% in clinical settings; 35.4% felt well prepared in classroom and 25.2% in clinical settings about treating TMD; 29.4% were confident in their TMD‐related knowledge. Students described their knowledge about risk factors (5‐point scale with 1 = *no knowledge*, Mean/SD = 4.20 ± 0.511) and psychosocial risk factors (Mean/SD = 3.63 ± 0.670), positively. They had positive TMD‐related attitudes, specifically that it is important to diagnose TMD (Mean/SD = 4.42 ± 0.658), that they felt responsible to treat patients with TMD (Mean/SD = 4.14 ± 0.754) and would manage TMD in their career (Mean/SD = 4.15 ± 0.756). They neither disagreed/nor agreed that they were comfortable selecting (Mean/SD = 2.90 ± 0.930) and performing diagnostic procedures (Mean/SD = 2.80 ± 0.937), recognizing TMD signs/symptoms (Mean/SD = 3.32 ± 0.909) and treating patients with TMD (Mean/SD = 2.82 ± 0.924). The more severe their own TMD‐related problems were, the more they knew about diagnosing and treating TMD (*r* = 0.11; *p* < 0.05) and risk factors (*r* = 0.15; *p* < 0.01), but the more they realized their own TMD‐knowledge related limitations (*r* = 0.13; *p* < 0.01). The more TMD‐related experiences they had, the more they educated themselves about TMD (*r* = 0.13; *p* < 0.05), the more motivated they were to learn more about it (*r* = 0.15; *p* < 0.01), the more they knew about risk factors (*r* = 0.17; *p* < 0.001), the more positive were their attitudes (*r* = 0.13; *p* < 0.01) and the more positively they evaluated treating patients with TMD (*r* = 0.19; *p* < 0.001).

**Conclusions:**

Dental education about TMD has to be improved. Personal TMD experiences are positively associated with TMD‐related educational motivation, knowledge, attitudes, and behavior.

## 1 Introduction

In 2024, a meta‐analysis of 74 studies published between January 1, 1994 and December 31, 2022, found that the incidence of TMD in the world population was 34% [1]. This percentage by far exceeded the first prevalence estimate of 6%–12% for TMD, published in 1992 [2]. The International Classification of Orofacial Pain [3] described TMD as a group of painful and non‐painful disorders affecting the temporomandibular joint (TMJ), muscles of mastication, and associated structures [2]. Research showed that patients with TMD often struggled to find healthcare providers and the providers consulted ranged widely [4]. In addition, patients with TMD were 10%–20% more likely to utilize general dental services than those without TMD [5]. In 2015, Horst et al. found that one in six dental patients reported orofacial pain within the last year, with pain in the muscles and TMJ being as frequent as dental and gingival pain [6]. In 2022, Grunberg et al. reported that treatment outcomes varied based on patient–provider interactions, with some patients describing these interactions as “diagnosed and dismissed,” “most providers don't have a clue,” and “it was a failure from the start” [7].

Given the high prevalence of TMD and lack of coordinated care, it is important to optimally educate future dental care providers about TMD‐related diagnosis and treatment. A first survey of 45 dental schools about curriculum coverage of TMD in 1973 found fragmented and inconsistent teaching, and a lack of standardization and time spent on this topic [8]. When Klasser and Greene surveyed U.S. and Canadian dental schools in 2007, they found a more integrated approach to TMD education, including education about comorbidities, psychological risk factors, and etiological considerations [9]. However, they still concluded that inadequacies remained in the TMD‐coverage in pre‐doctoral dental curricula [9]. This conclusion is interesting, given that the first comprehensive curriculum guidelines for TMD and Orofacial Pain for U.S. pre‐doctoral programs were published already in 1992 [10].

Most recently in 2022, the Commission on Dental Accreditation (CODA) implemented the Standard that added “graduates must be competent in providing oral health care within the scope of general dentistry, as defined by the school, including, temporomandibular disorders,” without further detail [11]. While this change requires that graduates of pre‐doctoral programs must be competent in this topic area, individual schools can determine how, when and what to teach. Insights into what should be covered was provided in 2021, when the American Academy of Orofacial Pain published new curriculum recommendations and guidelines for pre‐doctoral dental education programs. They described the following five domains, 1) Knowledge Base; 2) Screening, Evaluation, Diagnosis, and Risk Assessment; 3) Health Promotion and Prevention; 4) Clinical Decision‐Making, Treatment Planning, Evidence‐Based TMD Management, Communication, and Interdisciplinary Collaboration; and 5) Practice Management and Informatics [12]. Concerning didactic considerations, Klasser et al. stressed in 2023, that classroom‐based teaching should be augmented with exposure to live patient cases [13]. These authors argued that this approach is necessary to ensure that students grasp basic concepts, develop confidence in their patient care and are not limited in their TMD‐related knowledge. This argument points to the significance of raising students’ awareness for the significant impact TMD has on patients’ quality of life and well‐being.

Most recently, in 2025, Sangalli et al. published the results of a survey with deans/associate deans of academic affairs at 33 of the 72 CODA‐accredited U.S. pre‐doctoral dental school programs. They found that the majority of instruction was provided as formal teaching, with only 22.5% being based on clinical patient care. They also reported that 9.1% of the programs had no student clinical exposure to patients with TMD. Overall, they concluded that TMD instruction varied widely across different dental school programs, but that positive changes were found [14].

Concerning students’ self‐reported TMD‐related knowledge, a first study in 1990 showed that a comprehensive orofacial pain course in the dental curriculum resulted in 82% of students reporting that the course was helpful/extremely helpful and resulted in improved diagnostic skills [15]. However, a more recent study in 2023 based on electronic record reviews found that the majority of dental students failed to assess and often under‐diagnosed TMD, with students completing TMD assessments at only 11% of dental visits, despite 84% of patients reporting signs of TMD on their intake forms [16].

Research concerning practicing dentists’ attitudes showed that most practitioners had a fair, but not positive attitude towards treating patients with TMD [17, 18]. Overall, research concerning dental students’ TMD‐related attitudes has been missing. Given that attitudes are associated with behavior [19], it would be helpful to gain a better understanding of dental students’ attitudes and their relationships with TMD‐related educational motivation and behavioral intentions.

Considering the high prevalence of TMD in North America [1], it is likely that dental students themselves have personal TMD‐related experiences or have experiences with their family members, friends, or acquaintances. In 2022, Bal et al. reported that 46.4% of surveyed dental students had at least one TMD‐related symptom [20]. Most recently in 2024, Larkin et al. found that 90.1% of surveyed dental students reported at least one symptom of TMD [21]. So far, no research explored if students’ TMD‐related experiences were associated with increased interest and educational motivation in this content area. However, Marti et al., showed that personal experiences with an oral maxillofacial surgical (OMS) procedure were associated with an increased interest in OMS‐careers [22]. Therefore, it was hypothesized that students’ motivation for TMD‐related education would be associated with their own personal experiences as well as with observations of family members, friends, and acquaintances with TMD.

The objectives of this study were to assess dental students’ TMD‐related educational experiences, knowledge, attitudes, and behavior and to explore if having personal experiences themselves as well as with family members, friends, and acquaintances was positively associated with the constructs of interest.

## 2 Methods

This research was determined to be exempt from Institutional Review Board (IRB) oversight by the Health Sciences and Behavioral Sciences IRB at the University of Michigan, Ann Arbor, MI, USA, (#HUM00238051) on December 14, 2023. No written consent from the participants was required because responding to an online anonymous survey was considered as giving implicit consent to participate. This research was a cross‐sectional survey‐based study.

### 2.1 Respondents

To estimate the sample size needed to test for the significance of correlations between TMD‐related personal experiences, education, knowledge, attitudes, and behavior, an a priori power analysis with the G3.1.3. Power Analysis Program (http://www.psycho.uni‐duesseldorf.de/abteilungen/aap/gpower3/) was conducted. Assuming one‐sided hypotheses, a small to medium effect size of |*ρ*| = 0.16, an alpha error probability of 0.05, and a power of 0.95, the results showed that 414 respondents would be required to have the power to test such hypotheses. The one‐sided hypothesis was based on the objective that having personal experiences themselves as well as with family members, friends, and acquaintances would be positively associated with the constructs of interest. Expecting a certain number of responses with missing data, the goal was to collect data from at least 430 students. A total of 469 students at a Midwestern dental school participated in this research.

### 2.2 Procedure

Students received only one recruitment email that explained the purpose of the research and provided a web‐link/QR code for accessing an anonymous Qualtrics survey. At the same time, students in these classes were informed that they could respond to paper surveys and those paper surveys were handed out at the beginning of classes and returned anonymously to the research team at the end of the classes. The first survey response was submitted on January 16, 2024 and the last response on September 16, 2024.

### 2.3 Material

One dental researcher specialized in TMD and one survey methodologist collaborated on creating the first draft of the survey. They designed the survey based on the questions of interest. It consisted of five parts. Part 1 addressed the respondents’ background and educational characteristics. Table 1 provides a list of all the questions and answer alternatives for these questions. Part 2 asked about personal experiences with TMD, specifically about their own experiences with TMD and experiences with family members, friends, or acquaintances with TMD. These questions and answer alternatives are also provided in Table 1. Part 3 inquired about TMD‐related educational experiences. These questions were Likert Scale items with an answer scale ranging from 1 = *disagree strongly*, 2 = *disagree*, 3 = *neither disagree nor agree*, 4 = *agree*, 5 = *agree strongly*. The respondents used this answer scale to express their disagreement/agreement with the 16 statements provided in Table 2. Part 4 asked about TMD‐related knowledge. The 18 knowledge related items were also formulated as Likert scale items with the same five‐point answer scale used with the education‐related items. The wording of the eighteen statements is provided in Table 4. The final Part 5 consisted of attitudinal questions and items related to clinical activities. The wording of the 27 items covering this content area can be found in Table 5. The answer scale was the same five‐point Likert Scale that were used for the responses to the educational and knowledge‐related statements. The first draft of the survey was pilot tested with five students. Their feedback was used to finalize the survey.

### 2.4 Statistical Analysis

The data were collected with an anonymous web‐based survey provided on Qualtrics and with paper–pencil surveys. The web‐based responses were downloaded as an SPSS data file (IBM SPSS Statistics for Windows, Version 28.0. Armonk, NY, USA, IBM Corp). The paper‐based data were then entered into this file. Descriptive statistics such as frequency distributions, means, and standard deviations were computed to provide an overview of the responses to each of the individual items.

In addition, a factor analysis of the 14 educational items resulted in a three‐factor solution. Inter‐item consistency analyses showed that the items loading on each of the three factors had sufficiently high inter‐item consistency, with Cronbach alpha > 0.70 [23]. Three educational indices were therefore created by averaging the responses to the items loading on each factor respectively. A second factor analysis with the 18 TMD‐related knowledge items resulted in a four‐factor solution and a third factor analysis with the 23 attitudinal and clinical activity related items resulted also in a four‐factor solution. All three factor analyses used a Principal Component Analysis as the Extraction Method and a Varimax Rotation with Kaiser Normalization as the Rotation Method. Inter‐item consistency was determined with Cronbach alpha coefficients [22]. Indices were computed by averaging the responses to the items that loaded on each respective factor with an inter‐item consistency of > 0.70. Table 6 provide an overview of Pearson correlation coefficients that were used to analyze the associations with TMD‐related personal experiences and all the created indices. Because numerous statistical tests were performed simultaneously, a Bonferroni correction was applied, and the alpha value was set at *p* < 0.01 [24].

## 3 Results

### 3.1 Background Information and TMD‐Related Personal Experiences

Table 1 provides an overview of the background characteristics and the personal TMD‐related answers. Of the 469 students, 41.5% self‐identified as male and 58.3% as female, with one student reporting to be non‐binary. The average age was 23.77 years (Range 21–40 years). Half of the students were from European American backgrounds (50%), while 29.8% self‐identified as Asian American, 10.2% as Middle Eastern, 4.8% as African American, 1.5% as Hispanic/Latinx, and 3.7% as bi/multiracial. Students from all four academic years of the dental school curriculum were represented in the sample.

**TABLE 1:** Overview of the background characteristics and TMD‐related personal experiences of the dental student respondents.

Background characteristics	Frequencies *N* = 469	Percentages
Gender,		
Male	194	41.5%
Female	273	58.3%
Other	1	0.2%
Age	Mean = 23.77	SD = 2.096 Range, 21–40
Ethnicity/race,		
African American/Black	22	4.8%
Middle Eastern	47	10.2%
Asian American	137	29.8%
European American/White	230	50.0%
Hispanic/Latinx	7	1.5%
Bi‐/multi‐racial	17	3.7%

	Frequencies	Response rate,
Response rate, Started dental school in/were surveyed/dental class year		
6/2021–7/2024 = D4	67 of 121^a^, ^b^	55.4%
6/2022–9/2024 = D3	101 of 121	83.5%
6/2023–9/2024 = D2	95 of 109	87.2%
6/2023–1/2024 = D1	98 of 109	89.9%
6/2024–9/2024 = D1	98 of 109	89.9%
Total,	459 of 569	80.7%
Missing	10	

Personal experiences	Frequencies *N* = 469	Percentages
Do you have experiences with TMD yourself?	YES, 121	YES, 25.8%
Age when TMD first occurred?	Mean = 18.34	SD = 3.509 Range, 8–28
Did you receive an actual diagnosis of TMD?	YES, 38	YES, 8.1%
Severity of current TMD problem,		
1 = no problem	313	67.9%
2	81	17.6%
3	52	11.3%
4	15	3.3%
5 = severe problem	0	0%

Mean/SD	Mean = 1.82	SD = 1.253
Experiences with TMD,	YES,	YES
‐ a) In your family	134	28.7%
‐ b) Among friends	201	43%
‐ c) Among acquaintances	167	35.9%
‐ d) Among others	92	20.4%
‐ Others, patients	50	10.7%
Sum of personal experiences^c^	Mean = 1.24	SD = 1.224
Age of first hearing about TMD	Mean = 18.42	SD = 3.287 Range, 8–30

How did you first hear about it?	Frequencies YES	Percentages YES
Dental office related sources,		
From dentist/dental hygienist/specialist	63	13.4%
While working in dental office	40	8.5%
While shadowing a dentist	29	6.2%
Personal experiences,		
Own TMD‐related experiences	27	5.8%
From family members	57	12.2%
From friends	39	8.3%
From acquaintances	19	4.1%
Educational experiences,		
In dental school	57	12.2%
In college	16	3.4%
In high school	4	0.9%
Personal information search	18	3.8%

^a^ The frequencies of respondents were displayed as the number of respondents of the total number of students in each cohort.

^b^ The class sizes differ because at the beginning of the Winter term of the D2 year, students begin in the Internationally Trained Dentist Program

^c^ The “Sum of personal experiences” Score was computed by adding one point for each “Yes” responses to items a to d.

Approximately, 25.8% of the students reported having personal experiences with TMD (Mean onset year = 18.34 years; Range 8–28 years). Only 8.1% had received a formal TMD diagnosis. When asked how severe their TMD problems were, the answers ranged from “no problem” (67.9%), “slight problems” (17.6%), “some problems” (11.3%), to “problems” (3.3%). 28.7% reported they had TMD‐related experiences among family members, 43% among friends, 35.9% among acquaintances, and 20.4% among others, including 10.7% with patients. The age that they had first heard about TMD ranged from 8 to 30 years, with an average of 18.42 years.

A total of 28.1% had heard about TMD from dental office related sources such as from dentists, dental hygienists, or dental specialists (13.4%), while working in a dental office (8.5%), or while shadowing a dentist (6.2%). Overall, 30.4% had first heard about TMD because of personal experiences. In addition, 12.2% had first heard about TMD in dental school, 3.4% in college, 0.9% in high school, and 3.8% when personally searching for information.

### 3.2 TMD‐Related Educational Experiences

Table 2 provides an overview of responses related to educational experiences with TMD. Approximately, 49.9% agreed/agreed strongly that their dental school had prepared them well in diagnosing TMD in classroom‐based settings and 35.6% in clinical settings; 35.4% reported that their dental school had prepared them well to treat TMD in classroom‐based settings and 25.2% in clinical settings. The majority (83.9%) had learned about TMD in didactic courses, 38.5% in problem‐based learning, but only 29.5% were satisfied with their dental school education about TMD. Overall, the average “TMD‐related education in dental school” Index (mean = 3.32) showed that the students neither agreed nor disagreed that their TMD‐related dental education was positive.

**TABLE 2:** Percentages or responses and average TMD‐related educational experiences.

My dental school prepared me well in TMD diagnosis,	1^a^	2	3	4	5	Mean SD
a) In classroom‐based education.	0.4%	9.9%	39.8%	45.4%	4.5%	3.44 0.749
b) In clinical settings.	1.1%	12.0%	51.3%	32.0%	3.6%	3.25 0.753

**My dental school prepared me well in TMD treatment,**	**1** ^a^	**2**	**3**	**4**	**5**	**Mean** **SD**
c) In classroom‐based education.	2.2%	11.4%	51.1%	31.3%	4.1%	3.24 0.789
d) In clinical settings.	2.8%	14.3%	57.6%	21.7%	3.5%	3.09 0.779

**I have learned about TMD in,**	**1** ^a^	**2**	**3**	**4**	**5**	**Mean** **SD**
e) Didactic courses	0.4%	2.8%	12.8%	64.0%	19.9%	4.00 0.692
f) Problem based learning	4.3%	22.4%	34.8%	32.7%	5.8%	3.13 0.969
g) I am satisfied with my dental school education in TMD.	2.8%	18.8%	48.8%	25.9%	3.6%	3.09 0.836
**“TMD‐related dental school education” Index** ^b^ **(Cronbach alpha = 0.862)**	**Mean = 3.32**		**SD = 0.593**		**Range, 1.14–5.00**	

**Individual TMD‐related education**	**1** ^a^	**2**	**3**	**4**	**5**	**Mean** **SD**
h) Team based learning	6.2%	29.0%	34.3%	26.8%	3.6%	2.93 0.974
i) Clinic observations	3.4%	14.3%	33.1%	41.2%	7.9%	3.36 0.939
j) My clinical activities	3.6%	15.5%	172 36.9%	36.7%	34 7.3%	3.29 0.938
k) Other ways	11.4%	42 10.9%	236 61.0%	51 13.2%	14 3.6%	2.87 0.908
**“Individual TMD‐related education” Index** ^c^ **(Cronbach alpha = 0.742)**	**Mean = 3.09**		**SD = 0.693**		**Range, 1.00–5.00**	

**TMD‐related educational motivation**	**1** ^a^	**2**	**3**	**4**	**5**	**Mean** **SD**
l) I wish my dental school had educated me more in depth about TMD.	0.6%	3.9%	30.6%	49.9%	15.0%	3.75 0.778
m) I wish I had more clinical experiences in TMD in dental school.	0.4%	2.4%	26.1%	51.2%	19.9%	3.88 0.761
n) I want to learn more about TMD.	0.9%	1.7%	19.3%	49.7%	28.5%	4.03 0.790
**“Educational motivation” Index** ^d^ **(Cronbach alpha = 0.818)**	**Mean = 3.89**		**SD = 0.665**		**Range, 1.00–5.00**	

^a^ Answers ranged from 1 = *disagree strongly*, 2 = *disagree*, 3 = *neither disagree nor agree*, 4 = *agree* to 5 = *agree strongly*.

^b^ The “TMD‐related education in dental school” Index was created by averaging the responses to items a to g.

^c^ The “Individual TMD‐related education” Index was created by averaging the responses to items h to k.

^d^ The “Educational motivation” Index was created by averaging the responses to items l to n.

When asked about individual TMD‐related education, 30.4% had learned about TMD in team‐based learning, 49.1% in clinical observations, 44% during their clinical activities, and 16.8% in other ways. The “Individual TMD‐related Education” Index ranged from strongly disagreeing to strongly agreeing (Mean = 3.09). The last educational questions focused on students’ TMD‐related motivation to learn more about this topic. The majority wished their dental school had educated them more in depth about TMD (64.9%), that they had more clinical experiences in dental school (71.1%) and wanted to learn more about TMD (78.2%).

The students also answered an open‐ended question concerning how they had first heard about TMD (see Table 3). About three in 10 respondents first heard about it through personal experiences, or experiences of family members, friends, and acquaintances with TMD (30.3%). Approximately the same percentage (29.2%) had heard from dental office related sources such as from their dentist, dental hygienist, or dental specialist, or through shadowing or working in a dental office. In addition, 27.5% had first heard about TMD through one of eight educational experiences that are listed in Table 3.

**TABLE 3:** Frequencies and percentages of open‐ended responses concerning the sources of information about TMD.

Personal experiences	Frequencies	Percentages
Own TMD/heard when diagnosed/custom bite guard tx	27	5.8%
From family member/significant other with TMD	23	4.9%
Friends with TMD	22	4.7%
Information from parents/family members	19	4.1%
Information from friends	17	3.6%
Information from family members who work in healthcare	15	3.2%
From others/acquaintances with TMD experiences	11	2.4%
Information from others/acquaintances/peers	8	1.7%
**Sub total**	**142**	30.3%

**Information from dental office related sources**	**Frequencies**	**Percentages**
From dentist/dental hygienist/dental specialist	67	14.3%
From shadowing a dentist	29	6.2%
Working in dental office/health care setting	20	4.3%
Working as dental assistant	15	3.2%
Dental office	6	1.3%
**Sub total**	**137**	29.2%

**Educational experiences**	**Frequencies**	**Percentages**
Dental school/class in dental school	84	17.9%
Web search about TMD, podcast, social media	17	3.6%
Class in college, guest speaker in college	16	3.4%
High school classes	5	1.1%
Dental related event	2	0.4%
Discussion	2	0.4%
Reading about it	2	0.4%
Teacher	1	0.2%
**Sub total**	**129**	27.5%

**Other**	**Frequencies**	**Percentages**
Don't remember	8	1.7%
**Total # of respondents with first answer**	**367**	78.3%
**Total with second answer**	**46**	9.8%
**Total with third answer**	**2**	0.4%
**Total number of responses**	**415**	88.7%

### 3.3 TMD‐Related Knowledge

Table 4 provides an overview of the students’ TMD‐related knowledge answers. The majority agreed/agreed strongly (59.4%) that TMJ clicking is a serious symptom of TMD, that TMD pain is aggravated by jaw motion (84.6%), that occlusal splint use is a good therapy for patients with TMD (69%), and that occlusal management is helpful for treating TMD (76.1%). The majority also agreed/agreed strongly with statements about risk factors for TMD, that TMD is a physical anatomical problem (93.3%) and that stress (92.8%) are factors in TMD development. Responses to questions concerning the role of psychosocial risk factors showed that 67.8% agreed/agreed strongly that TMD is a behavioral problem, 61.5% that TMD is a psychosocial problem, and 59.6% that depression is an important etiologic factor in TMD. However, 31.2% wrongly agreed/agreed strongly that occlusal splints eliminate bruxism.

**TABLE 4:** Percentages of responses and average knowledge related to TMD.

Knowledge related responses^a^, Diagnosis and treatment of TMD	1^b^	2	3	4	5	Mean SD
a) TMJ clicking is a serious symptom of TMD.	3.4%	11.6%	25.5%	45.9%	13.5%	3.55 0.979
b) TMD pain is aggravated by jaw motion.	0.0%	0.2%	15.2%	63.4%	21.2%	4.06 0.609
c) Occlusal splint use is a good therapy for patients with TMD.	0.6%	1.7%	28.7%	55.9%	13.1%	3.79 0.708
d) Occlusal management is helpful for treating TMD.	0.4%	2.6%	20.9%	63.8%	12.3%	3.85 0.673
**“Diagnosis and treatment of TMD” Index** ^c^ **(Cronbach alpha = 0.667)**	**Mean = 3.81**		**SD = 0.533**		**Range, 2.25–5**	

**Knowledge related responses, Risk factors for TMD**	**1** ^a^	**2**	**3**	**4**	**5**	**Mean** **SD**
e) TMD is a physical/anatomical problem.	0.0%	1.1%	5.6%	67.9%	25.4%	4.18 0.567
f) Stress is a factor in the development of TMD.	0.0%	0.9%	6.4%	54.1%	38.7%	4.31 0.627
g) Oral parafunctional habits is a factor in the development of TMD.	0.0%	1.7%	13.1%	55.7%	29.6%	4.13 0.692
**“Risk factor” Index** ^c^ **(Cronbach alpha = 0.737)**	**Mean = 4.20**		**SD = 0.511**		**Range, 2–5**	

**Knowledge related responses, Psychosocial risk factors for TMD**	**1** ^a^	**2**	**3**	**4**	**5**	**Mean** **SD**
h) TMD is a behavioral problem.	1.7%	8.8%	21.6%	55.0%	12.8%	3.69 0.868
i) TMD is a psychosocial problem.	1.7%	8.4%	28.5%	51.0%	10.5%	3.60 0.849
j) Depression is an important etiologic factor in TMD.	0.9%	6.9%	32.8%	49.5%	10.1%	3.61 0.793
**“Psychosocial risk factors” Index** ^e^ **(Cronbach alpha = 0.719)**	**Mean = 3.63**		**SD = 0.670**		**Range, 1–5**	

**Knowledge related responses, TMD‐related clinical responsibility**	**1** ^a^	**2**	**3**	**4**	**5**	**Mean** **SD**
k) It is important to me to be able to diagnose TMD.	0.0%	1.1%	6.2%	42.2%	50.5%	4.42 0.658
l) I feel responsible to treat patients with TMD.	0.6%	1.5%	14.1%	51.1%	32.7%	4.14 0.754
m) I will manage TMD cases in my career.	0.4%	1.9%	13.9%	50.0%	33.8%	4.15 0.756
**“TMD‐related clinical responsibility” Index** ^f^ **(Cronbach alpha = 0.737)**	**Mean = 4.24**		**SD = 0.587**		**Range, 2.67–5**	

**Knowledge related responses,** **TMD‐related clinical expertise**	**1**	**2**	**3**	**4**	**5**	**Mean** **SD**
n) I recognize my own limitations with managing TMD.	0.2%	1.7%	17.6%	52.0%	28.5%	4.07 0.738
o) I recognize my limitations when treating patients with TMD.	0.2%	1.1%	14.0%	52.2%	32.5%	4.16 0.710
**“TMD‐related limitation” Index** ^g^ **(Cronbach alpha = 0.670)**	**Mean = 4.11**		**SD = 0.628**		**Range, 1–5**	

**Single items**	**1** ^a^	**2**	**3**	**4**	**5**	**Mean** **SD**
p) I am confident in my knowledge concerning TMD.	7.9%	30.3%	32.4%	22.7%	6.7%	2.90 1.052
q) In 2020, TMD and Orofacial Pain gained specialty recognition.	0.2%	1.3%	40.0%	39.6%	18.9%	3.76 0.776

^a^ All statements from a–j are considered are included based on provided references [3, 43, 44].

^b^ The answers ranged from 1 = *disagree strongly*, 2 = *disagree*, 3 = *neither disagree nor agree*, 4 = *agree* to 5 = *agree strongly*.

^c^ The “Diagnosis and treatment of TMD” Index was created by averaging the answers to items a–d.

^d^ The “Risk factor” Index was created by averaging the answers to items e–g.

^e^ The “Psychosocial risk factors” Index was created by averaging the answers to items h–j.

^f^ The “TMD‐related clinical responsibility” Index was created by averaging the answers to items k–m.

^g^ The “TMD‐related limitation” Index was created by averaging the answers to items n and o.

Concerning TMD‐related clinical responsibilities, the absolute majority (92.7%) agreed/agreed strongly that it was important to them to be able to diagnose TMD, that they felt responsible to treat patients with TMD (83.8%), and that they would manage TMD cases in their future career (83.8%). However, 80.5% recognized their own limitations with managing TMD and 84.7% with treating patients with TMD. Only 29.4% were confident in their TMD‐related knowledge. The majority (58.5%) knew that “TMD and Orofacial Pain” had gained specialty recognition status in 2020[25].

### 3.4 TMD‐Related Attitudes and Professional Behavior

When asked if working with patients with TMD is interesting, satisfying, and rewarding, the students agreed on average (5‐point scale with 5 = *agree strongly*, Mean = 3.88) (See Table 5). In addition, when asked how much working with patients with TMD is frustrating, stressful, and difficult, the average response was between “*neither disagree nor agree*” and “*agree*” (Mean = 3.46).

**TABLE 5:** Percentages of and average responses related to students’ attitudes and clinical activities concerning TMD.

Positive TMD‐related patient experiences, Working with patients with TMD is,	1^a^	2	3	4	5	Mean SD
a) Interesting	0.2%	3.2%	20.4%	53.1%	23.0%	3.95 0.763
b) Satisfying	1.1%	3.7%	32.5%	46.2%	16.6%	3.74 0.815
c) Rewarding	0.0%	2.8%	24.8%	47.8%	24.6%	3.94 0.777
**“Positive patient experience” Index** ^b^ **(Cronbach alpha = 0.842)**	**Mean = 3.88**		**SD = 0.685**		**Range, 1.33–5**	

Negative TMD‐related patient‐experiences, Working with patients with TMD is,	1^a^	2	3	4	5	Mean SD
d) Frustrating	3.7%	14.0%	40.1%	35.3%	6.9%	3.28 0.917
e) Stressful	1.1%	9.7%	46.8%	34.7%	7.8%	3.38 0.807
f) Difficult	0.2%	3.2%	35.7%	46.0%	14.8%	3.72 0.760
**“Negative patient experience” Index** ^c^ **(Cronbach alpha = 0. 742)**	**Mean = 3.46**		**SD = 0.675**		**Range, 1–5**	

Comfortability with clinical activity related statements	1^a^	2	3	4	5	Mean SD
g) Performing clinical/physical examinations of TMD.	4.5%	17.1%	32.3%	39.7%	6.4%	3.26 0.968
h) Select diagnostic procedures in TMD.	6.7%	25.8%	41.6%	23.0%	3.0%	2.90 0.930
i) Perform diagnostic procedures in TMD.	7.7%	29.5%	40.6%	19.2%	3.0%	2.80 0.937
j) Interpret diagnostic procedures in TMD.	7.5%	28.2%	38.9%	23.7%	1.7%	2.84 0.929
k) Diagnosing patients with TMD.	8.4%	27.3%	38.6%	22.5%	3.2%	2.85 0.970
l) Evaluating psychological and behavioral factors of TMD.	4.7%	19.0%	37.4%	35.5%	3.4%	3.14 0.923
m) Managing psychological and behavioral factors of TMD.	6.0%	24.1%	40.4%	26.5%	3.0%	2.96 .931
n) Treating patients with TMD.	7.9%	27.8%	40.9%	21.4%	1.9%	2.82 0.924
o) Applying pharmacology therapy for TMD.	13.3%	36.4%	36.0%	12.4%	1.9%	2.53 0.938

Comfortability with clinical activity related statements, Continued	1^a^	2	3	4	5	Mean SD
p) Apply jaw exercise therapy for TMD.	8.8%	33.5%	34.8%	19.4%	3.4%	2.75 0.979
q) Making behavioral management recommendations.	6.2%	22.5%	36.1%	31.5%	3.6%	3.04 0.968
r) Managing patients with intraoral occlusal appliances.	8.3%	22.2%	35.3%	30.6%	3.6%	2.99 1.004
s) Educating patients about TMD.	5.1%	17.9%	33.3%	37.6%	6.0%	3.21 0.978
t) Discussing cases in a multidisciplinary team.	5.8%	19.4%	35.9%	33.3%	5.6%	3.13 0.982
**“Comfort with TMD‐related activities” Index** ^d^ **(Cronbach alpha = 0.965)**	**Mean = 2.95**		**SD = 0.784**		**Range, 1–5**	

TMD‐related patient management	1^a^	2	3	4	5	Mean SD
u) Recognizing of the signs and symptoms of TMD.	2.6%	17.8%	29.8%	45.0%	4.9%	3.32 0.909
v) Performing a relevant patient history.	1.5%	9.8%	21.4%	53.0%	14.3%	3.69 0.888
w) Referring to other providers.	2.8%	10.3%	26.7%	41.8%	18.3%	3.63 0.989
**“TMD‐related patient management” Index** ^e^ **(Cronbach alpha = 0.735)**	**Mean = 3.54**		**SD = 0.751**		**Range, 1–5**	

^a^ Answers ranged from 1 = *disagree strongly*, 2 = *disagree*, 3 = *neither disagree nor agree*, 4 = *agree* to 5 = *agree strongly*.

^b^ The “Positive patient experience” Index was created by averaging the answers to items a–c.

^c^ The “Negative patient experience” Index was created by averaging the answers to items d–f.

^d^ The “Clinical TMD‐related activities” Index was created by averaging the answers to items g–t.

^e^ The “TMD‐related patient management” Index was created by averaging the answers to items u–w.

A total of 14 questions inquired how comfortable the students were with clinical TMD‐related activities. Less than half of the respondents were comfortable with diagnostic activities. For example, 46.1% were comfortable performing clinical physical examinations of TMD, 26% were comfortable with collecting TMD‐related diagnostic procedures, 22.2% with performing diagnostic procedures, and 25.4% with interpreting diagnostic procedures. Only 25.7% were comfortable with diagnosing TMD, 38.9% with evaluating psychological and behavioral factors, and 29.5% with managing psychological behavioral factors.

Concerning the treatment of patients with TMD, approximately one in four respondents (23.3%) were comfortable with treating patients with TMD, 14.3% with applying pharmacology therapy for TMD, 22.8% with applying jaw exercise therapy for TMD, 34.2% with managing patients with intraoral occlusal appliances, and only 38.9% were comfortable discussing cases in multidisciplinary teams. Concerning TMD‐related patient management, the responses showed that 49.9% were comfortable recognizing signs and symptoms of TMD, 67.3% with performing the relevant patient history and 60.1% with referring to other providers.

### 3.5 Relationship Between Constructs of Interest

A final objective focused on gaining a better understanding how these constructs of interest were interrelated. Table 6 provides an overview of the relationships between personal and educational experiences, and the dental students’ knowledge, attitudes and professional behavior. Concerning personal experiences, the more severe the students’ own TMD‐related experiences were, the more likely they were to also have had personal experiences with family members, friends, and acquaintances (*r* = 0.28; *p* < 0.001) and the younger they had been when they had first heard about TMD (*r* = −0.20; *p* < 0.001). In addition, the more family members, friends, and/or acquaintances with TMD they had, the more likely they were to know about TMD risk factors (*r* = 0.17; *p *< 0.001), the more they wanted to learn about TMD (*r* = 0.15; *p* < 0.01) and the more positive they described TMD‐related experiences with patients (*r* = 0.19; *p* < 0.001). The younger they were when they had first heard about TMD, the more likely they were to have family members, friends, and/or acquaintances with TMD (*r* = −0.21; *p* < 0.001) and the more positively they described TMD‐related experiences with patients (*r* = 0.19; *p* < 0.001).

**TABLE 6:** Correlations between personal experiences, TMD‐related education, knowledge, attitudes, and professional behavior.

	TMD‐related personal experiences			Education related experiences		
TMD‐related personal experiences	Severity of personal TMD^a^	Sum of personal experiences^b^	Age first heard^c^	Graduation year^d^	TMD dental school education^e^	Individual Education^e^
Severity of TMD^a^	1	0.28^***^	−0.20^***^	0.05	0.01	0.03
Sum of personal experiences^b^	0.28^***^	1	−0.21^***^	−0.03	0.02	0.13
Age first heard^c^	−0.20^***^	−0.21^***^	1	−0.15^**^	−0.02	−0.03

**TMD‐related educational experiences**	**Severity of personal TMD**	**Sum of personal experiences**	**Age first heard**	**Graduation year**	**Dental school education**	**Individual education**
Graduation year^d^	0.05	−0.03	−0.15^**^	1	−0.07	−0.12
Patient experiences^e^	−0.00	0.29^***^	0.10	−0.19^***^	−0.03	0.01
TMD‐related dental school education^e^	0.01	0.02	−0.02	−0.07	1	0.68^***^
Individual education^e^	0.03	0.13	−0.03	−0.12	0.68^***^	1

**TMD‐related knowledge about**	**Severity of personal TMD**	**Sum of personal experiences**	**Age first heard**	**Graduation year**	Dental school education	**Individual education**
Diagnosis/treatment^g^	0.11	0.05	−0.08	0.28^***^	0.28^***^	0.24^***^
Risk factors^g^	0.15^**^	0.17^***^	−0.06	−0.07	0.25^***^	0.17^***^
Behavioral Risk factors^g^	0.04	0.14^**^	−0.11	−0.09	0.25^***^	0.26^***^
Clinical responsibility^g^	0.08	0.13^**^	−0.11	0.20^***^	0.17^***^	0.10
Limitations^g^	0.13^**^	0.10	0.07	−0.25^***^	0.17^***^	0.08

**TMD related attitudes**	**Severity of personal TMD**	**Sum of personal experiences**	**Age first heard**	**Graduation year**	Dental school education	**Individual education**
Educational motivation^e^	0.07	0.15^**^	−0.03	−0.12^**^	−0.01	−0.02
Positive patient experiences^g^	0.07	0.19^***^	−0.17^***^	0.06	0.15^**^	0.13^**^
Negative patient experiences^g^	0.05	0.10	−0.03	−0.21^***^	0.05	0.18^***^

**TMD‐related professional behavior**	**Severity of personal TMD**	**Sum of personal experiences**	**Age first heard**	**Graduation year**	Dental school education	**Individual education**
Comfortable with clinical work^d^	0.07	0.05	−0.09	−0.17^***^	0.48^***^	0.46^***^
TMD management^d^	0.07	0.10	−0.01	−0.29^***^	0.43^***^	0.32^***^

^a^ The severity of the respondents’ current TMD problem was assessed on a scale from 1 = “No problem” to 5 = “Severe problem.”

^b^ The “Sum of personal experiences” Score was computed by adding one point for each “Yes” response to items a–d.

^c^ The answer to the “Age first heard” question was to provide the age the respondents first heard about TMD.

^d^ The “Graduation year” question referred to the future date at which the students expected to graduate from dental school.

^e^ The information about how these indices were constructed can be found in the Legend to Table 2.

^f^ The information about how these indices were constructed can be found in the Legend to Table 4.

^g^ The information about how these indices were constructed can be found in the Legend to Table 5.

^***^ *p* < 0.001.

^**^ *p* < 0.010

^*^ *p* < 0.05.

Concerning the relationships between educational experiences and the students’ knowledge, attitudes, and clinical behavior, the data showed that the longer the students had attended dental school, the more knowledge they had about TMD‐related diagnosis and treatment (*r* = 0.28; *p* < 0.001), and the more they perceived to have TMD‐related clinical responsibilities (*r* = 0.20; *p* < 0.001). In addition, the closer they were to graduation, the less they felt they lacked expertise (*r* = −0.25; *p* < 0.001), the fewer negative patient experiences they had (*r* = −0.21; *p* < 0.001), the more comfortable they were working in clinical settings with TMD (*r* = −0.17; *p* < 0.001) and the more positive TMD‐related patient management they had (*r* = −0.29; *p* < 0.001).

The quality of the dental school education correlated positively with their personal education outside of the dental school setting (*r* = 0.68; *p* < 0.001) and with TMD‐related knowledge about diagnosis/treatment (*r* = 0.28; *p* < 0.001), risk factors (*r* = 0.25; *p* < 0.001), behavioral risk factors (*r* = 0.25; *p* < 0.001), and their clinical responsibility (*r* = 0.17; *p* < 0.001). It also correlated positively with how comfortable they were with TMD‐related clinical work (*r* = 0.48; *p* < 0.001) and patient management (*r* = 0.43; *p* < 0.001). The personal education correlated positively with the TMD‐related diagnosis/treatment knowledge (*r* = 0.24; *p* < 0.001), risk factor knowledge (*r* = 0.17; *p* < 0.001), behavioral risk factor knowledge (*r* = 0.26; *p* < 0.001), negative patient experiences (*r* = 0.18; *p* < 0.001), the degree to which they were comfortable with clinical work (*r* = 0.46; *p* < 0.001), and with TMD‐related management (*r* = 0.32; *p* < 0.001).

## 4 Discussion

The high prevalence of TMD [1] inflicts a cost of around $4 billion annually on the United States (U.S.) economy [26], and the finding that 16% of TMD sufferers did not respond to standard treatments show the great need for education of future dental care providers [27]. Specific gaps in TMD‐related education were highlighted in a 2020 report from the National Academies of Sciences, Engineering and Medicine calling for improving the information related to reducing TMD‐related pain and suffering [28]. Considering these reported educational gaps [28] led to the question which factors might be associated with dental students’ TMD‐related knowledge, attitudes, and professional behavioral intentions. The main factors considered here were students’ own experiences with TMD as well as their experiences with family members, friends and acquaintances with TMD. The decision to consider these personal experiences was influenced by Klasser et al. [13] who suggested in their 2023 publication that including patient cases could be helpful in TMD‐related training. The resulting research question therefore was whether personal experiences with TMD as well as experiences with TMD among family members, friends, and acquaintances would be equally or even more influential in increasing educational motivation knowledge, and improving TMD‐related attitudes and behavioral intentions.

Approximately a quarter of the respondents reported that they themselves had TMD symptoms or even a TMD diagnosis. This percentage exceeded the 1993 estimate that 6%–12% of the U.S. population had TMD [2], but was lower than the most recent estimate in 2021 of 31.1% in the U.S. population [29]. However, the finding that the average age of TMD onset ranged from 8–28 years of age might explain why these dental students with an average age under 24 years might have had a lower percentage than was reported for the U.S. population.

In addition, the ethnicity/race of the respondents was not in parity with the U.S. data in 2024 [30], with a lower percentage of African American dental students and a higher percentage of Asian American respondents compared to the U.S. population. This fact is noteworthy because research showed that the prevalence of TMD was higher among African American children and adults than among non‐African American individuals [31, 32]. The disproportionately lower percentage of African American dental students in this study might have contributed to the overall lower percentage of students with TMD symptoms.

While large percentages of students had personal experiences with TMD and/or had family members, friends, or acquaintances with TMD and thus were likely to have first heard about TMD in this context, a substantial percentage of three in 10 students had first heard about TMD in dental offices and slightly more than one in 10 students reported to have first heard about it in dental school. This wide range of how students had first heard about TMD and then received additional TMD‐related information provides the opportunity to explore the objectives of interest in this research, namely if having personal experiences were associated with increased educational motivation and knowledge and more positive attitudes and behavioral intentions.

Overall, students’ perceptions of their educational experiences showed that more learning had occurred in classroom‐based settings than in clinical settings. This finding was consistent with previous research reported by Gonzalez et al. [33]. These authors found that a dental school elective for dental students with “hands‐on” clinical TMD‐related experiences resulted in a significant improvement of these students’ competency in TMD diagnosis and treatment [33]. However, in 2012, dental students still reported that their orofacial pain training was minimal and that they did not have adequate knowledge in clinical practice [34]. In 2015, Teich et al. also found that dental students wanted to learn more about TMD in clinical scenarios [35]. The fact that the most common teaching modality for TMD‐related material was didactic lectures and that less than half of students interacted with live patients was also discussed by Breckons et al. [36]. These authors reported that integrating patient interactions into dental student educational experiences led to more confidence in TMD clinical management [36]. In 2020, Costa et al. showed that patient interactions during training resulted in early detection of TMD [37]. Based on the previous research and the findings in this study, integrating patient experiences early and throughout the curriculum is highly recommended.

Concerning the students’ TMD‐related knowledge the data showed that while the majority was knowledgeable about diagnosing and identifying risk factors, the majority still reported limitations when managing and treating patients with TMD and nearly four out of 10 students were not confident in their TMD knowledge. These findings were consistent with research published in 2023, when Tormes et al. reported that dental students were limited in recognizing TMD pathophysiology [38]. At the dental school were this survey was administered, TMD‐related material is woven through all four years of the curriculum. It is presented starting in Year 1 with classroom‐based education and includes clinical activities increasing from merely assisting in Year 1 to being engaged in clinical activities in dental school clinics, the TMD & Orofacial Pain Clinic and in community‐based education in Year 4. It is important to note that Table 6 shows how their standing in dental school is associated with all relevant TMD‐related constructs of interest. However, the fact that first and second year dental students participated in this current research might also explain the knowledge‐related findings. Previous research showed that comfort level and competency performance had improved over the course of dental school education [39, 40].

The results concerning the students’ TMD‐related attitudes showed that the absolute majority used positive adjectives to describe their experiences. This finding was consistent with the results by Vallon and Nilner who reported that 90% of dental students had used positive adjectives when describing their experiences with chronic orofacial pain patients [40]. However, students also described their experiences with TMD patients as difficult which might be indicative of their realization of the complexity of TMD.

One central objective of this research was to explore whether students’ own personal experiences as well as their experiences with family, friends, and acquaintances with TMD were associated with their TMD‐related educational experiences, knowledge, attitudes, and behavioral intentions. The results supported that personal experiences play an important role in this context. There is a strong association between having more severe TMD symptoms and TMD‐related knowledge, their ability to recognize their limitations, and their personal responsibility to treat patients with TMD in the future. Previous research showed that early experiences aided in developing appropriate attitudes toward patient care among health profession students [41]. More recently in 2017, Leadbetter et al. reported that oral health students’ personal and/or patient experiences resulted in improved learning motivation and greater connection to their profession [42]. The findings in this current study indicated that students’ personal experiences with TMD correlated with their motivation to learn more about TMD, their knowledge and attitudes and behavioral intentions. Assuring that dental students without personal TMD‐related experiences receive case‐based and hands‐on education with patients might support them in becoming more aware of the ways TMD affects patient's lives and the importance to be qualified to support these patients and provide the best possible care.

The most important and relevant lessons learned are connected with the finding that personal experiences are influential for students’ TMD‐related considerations. Encouraging faculty to use case studies and in‐depth patient encounters could be one way to increase students’ understanding of the complexity of this subject matter. Given the high prevalence of TMD diagnoses and the severe impact of TMD on patients’ lives, it is important to encourage CODA not to take away from TMD‐related requirements but to even strengthen them more. Integrating early patient‐based experiences in TMD classroom‐based and clinical settings will be beneficial for students’ educational motivation, knowledge, attitudes, and behavior.

This research has several limitations. First, one could argue that this research focused only on students in one dental school. However, it is important to realize that the main focus was to explore the impact of personal experiences and their relationships with learning outcomes. The wide range of personal experiences allowed investigation of this objective. Second, only 4.8% of the respondents self‐identified as African American. Given that TMD is more prevalent in African Americans [31, 32], future research should focus to include more African American students such as from historically Black dental schools to further explore the role of ethnicity/race in this context. Third, the fact that multidisciplinary treatment approaches were only considered with one question in this current research is limiting the understanding of this important component of TMD care. Future research should therefore focus on this topic. In addition, asking only closed‐ended questions about the students’ satisfaction with their TMD‐related education did not allow to explore the reasons for dissatisfaction adequately. Future research should include open‐ended questions to gain a better understanding how to make TMD‐training more optimal. Finally, TMD‐related knowledge and behavior were only assessed via self‐report. Future research should complement the self‐assessed findings with objective indicators. Clinical chart review data could be quite helpful in this context [16].

## 5 Conclusions

Based on these findings, it can be concluded that the majority of dental students are not satisfied with their TMD‐related education. They report to be better educated in classroom based than in clinical settings. While students lack confidence in TMD‐related knowledge, they have positive TMD‐related attitudes and feel responsible for diagnosing and managing TMD. Selecting and performing diagnostic procedures, recognizing signs and symptoms, and treating TMD are areas for needed growth in the pre‐doctoral dental curriculum as students neither agree nor disagree that they feel comfortable in these areas.

Personal TMD‐related experiences are associated with students’ perceptions of the related education, their knowledge, attitudes, and behavioral intentions. The more severe their TMD‐related problems, the more knowledge they have about diagnosis, treatment, and risk factors and the better they are able to assess their limitations when treating patients with TMD. They are more likely to educate themselves when they, their family, friends, and/or acquaintances have signs and symptoms of TMD. Having personal experiences with TMD also correlates with motivation, positive attitudes, and knowledge. In summary, these findings indicate that both didactic and clinical experiences in TMD need to be improved in pre‐doctoral dental education, with a special focus on “hands‐on” experiences and introducing vivid case description and live patient encounters.
